# Coronary microvascular ischemia in hypertrophic cardiomyopathy - a pixel-wise quantitative cardiovascular magnetic resonance perfusion study

**DOI:** 10.1186/s12968-014-0049-1

**Published:** 2014-08-12

**Authors:** Tevfik F Ismail, Li-Yueh Hsu, Anders M Greve, Carla Gonçalves, Andrew Jabbour, Ankur Gulati, Benjamin Hewins, Niraj Mistry, Ricardo Wage, Michael Roughton, Pedro F Ferreira, Peter Gatehouse, David Firmin, Rory O’Hanlon, Dudley J Pennell, Sanjay K Prasad, Andrew E Arai

**Affiliations:** 1 Cardiovascular Magnetic Resonance and Cardiovascular Biomedical Research Units, Royal Brompton Hospital, London, UK; 2 Imperial College London, London, UK; 3 Advanced Cardiovascular Imaging Laboratory, National Heart, Lung, and Blood Institute, National Institutes of Health, Bethesda, Maryland, USA; 4 R-Squared Statistics, London, UK

**Keywords:** Hypertrophic cardiomyopathy, Perfusion, Cardiovascular magnetic resonance, Microvascular dysfunction, Sudden cardiac death

## Abstract

**Background:**

Microvascular dysfunction in HCM has been associated with adverse clinical outcomes. Advances in quantitative cardiovascular magnetic resonance (CMR) perfusion imaging now allow myocardial blood flow to be quantified at the pixel level. We applied these techniques to investigate the spectrum of microvascular dysfunction in hypertrophic cardiomyopathy (HCM) and to explore its relationship with fibrosis and wall thickness.

**Methods:**

CMR perfusion imaging was undertaken during adenosine-induced hyperemia and again at rest in 35 patients together with late gadolinium enhancement (LGE) imaging. Myocardial blood flow (MBF) was quantified on a pixel-by-pixel basis from CMR perfusion images using a Fermi-constrained deconvolution algorithm. Regions-of-interest (ROI) in hypoperfused and hyperemic myocardium were identified from the MBF pixel maps. The myocardium was also divided into 16 AHA segments.

**Results:**

Resting MBF was significantly higher in the endocardium than in the epicardium (mean ± SD: 1.25 ± 0.35 ml/g/min versus 1.20 ± 0.35 ml/g/min, P < 0.001), a pattern that reversed with stress (2.00 ± 0.76 ml/g/min versus 2.36 ± 0.83 ml/g/min, P < 0.001). ROI analysis revealed 11 (31%) patients with stress MBF lower than resting values (1.05 ± 0.39 ml/g/min versus 1.22 ± 0.36 ml/g/min, P = 0.021). There was a significant negative association between hyperemic MBF and wall thickness (β = −0.047 ml/g/min per mm, 95% CI: −0.057 to −0.038, P < 0.001) and a significantly lower probability of fibrosis in a segment with increasing hyperemic MBF (odds ratio per ml/g/min: 0.086, 95% CI: 0.078 to 0.095, P = 0.003).

**Conclusions:**

Pixel-wise quantitative CMR perfusion imaging identifies a subgroup of patients with HCM that have localised severe microvascular dysfunction which may give rise to myocardial ischemia.

## Background

Hypertrophic cardiomyopathy is a common inherited heart muscle disease which can lead to premature sudden cardiac death (SCD) or to progressive heart failure in a subset of patients [1]–[3]. Coronary microvascular ischemia has been implicated in the pathogenesis of replacement fibrosis [4],[5], which in turn has been associated with adverse outcomes [6]–[13]. However, myocardial replacement fibrosis is characteristically found in the mid-wall [14], whereas perfusion abnormalities are thought to preferentially affect the endocardium [15]. Varnava *et al*. found a poor correspondence between the distribution of small vessel disease and fibrosis on post-mortem histology [16]. The precise relationship between fibrosis and myocardial ischemia therefore remains unresolved.

Cardiovascular magnetic resonance (CMR) allows the non-invasive assessment of myocardial blood flow and replacement fibrosis without the use of ionising radiation [17]. Only one study to date has utilised multi-parametric CMR to quantitatively evaluate the relationship between perfusion abnormalities, left ventricular (LV) wall thickness, and fibrosis [18]. In keeping with previous studies that used positron emission tomography (PET) for the absolute quantification of myocardial blood flow (MBF), this study assessed perfusion using a sector-based approach [19]-[22]. This strategy creates territories that are of relevance for assessing epicardial coronary disease but which are arbitrary from a microcirculatory perspective. The use of a sector-based or global approach alone whilst improving signal-to-noise, may mask or significantly underestimate the local severity of perfusion defects due to coronary microvascular dysfunction [23]. This is of particular importance as the burden and severity of ischemia may be of prognostic importance as a mechanism leading to myocardial fibrosis, and independently as a trigger for ventricular arrhythmia [19],[24].

We have previously shown that first-pass gadolinium-enhanced CMR stress perfusion imaging allows the pixel-wise quantification of absolute MBF with high fidelity and spatial resolution [23]. We applied these pixel-level techniques to explore the severity of perfusion abnormalities in HCM and to assess their relationship to fibrosis and local wall thickness.

## Methods

### Study population

Thirty-six patients with HCM referred for CMR at the Royal Brompton Hospital were studied. HCM was diagnosed in accordance with standard clinical guidelines [25]. Patients were excluded if they had: conditions associated with coronary microvascular dysfunction such as diabetes; significant epicardial coronary artery disease on angiography (defined as >50% diameter stenosis in a major coronary artery); previous gradient reduction therapy; contraindications to CMR, adenosine, or gadolinium-based contrast agents. The study was approved by the National Research Ethics Service and was conducted in accordance with the principles set out in the declaration of Helsinki, with written informed consent obtained from all patients.

### Image acquisition

All patients were asked to abstain from caffeine-containing beverages or drugs for 24 hours prior to imaging and from β-blockers and rate-limiting calcium channel antagonists for 48 hours prior to imaging. Images were acquired using a dedicated 1.5 T scanner with a twelve-channel phased-array receiver coil (Siemens Magnetom Avanto, Siemens AG Healthcare Sector, Erlangen, Germany). A retrospectively-gated balanced steady-state free-precession sequence was used to obtain breath-hold cine images in three long-axis planes, followed by a contiguous stack of short axis slices from the atrioventricular ring to the apex [26]. The end-systolic frames of the long-axis cine images were used to plan the acquisition of three short axis perfusion images to cover the base, mid-LV and the apex. Adenosine was infused at 140 mcg/kg/min for a minimum of 4 minutes to achieve hyperemia. After measurement of heart rate and blood pressure at peak stress, gadolinium contrast (Gadovist, Bayer-Schering, Berlin, Germany, 0.1 mmol/kg) was rapidly injected at 3.5 ml/s, followed by 15 ml saline at 7 ml/s using a power injector (Medrad UK, Ely, Cambridgeshire, UK) to ensure a compact bolus entered the heart. A saturation-recovery prepared dual-sequence approach with center-out hybrid echoplanar imaging (EPI) [27] was used for perfusion imaging with the following typical sequence parameters: fat saturation pulse, composite 90° saturation preparation pulse for each slice [28], 28° readout pulse, saturation recovery time to central raw data acquisition 90 ms, repetition time 5.1 ms, echo time 1.1 ms, echo train length 4, field of view 360 × 288 mm, base resolution 160 × 160, slice thickness 8 mm. The center frequency of the scanner electronics was manually tuned to water in a 10 × 10 × 10 cm volume encompassing the LV to optimise both fat suppression and center-out hybrid-EPI image quality [29]. The arterial input function slice used low-resolution fast low-angle shot (FLASH) imaging with an adiabatic B1-insensitive rotation type 4 saturation pulse [30].

Three short axis images and an image at the basal slice of the arterial input function were acquired every cardiac cycle for a minimum of 50 cycles. Two initial proton density-weighted images were acquired prior to the arrival of contrast as part of perfusion imaging and were used for subsequent surface coil intensity correction. After ~10 min, late gadolinium enhancement (LGE) images were acquired with an inversion recovery-prepared segmented turbo FLASH sequence [31]. Inversion times were optimised to null normal myocardium with images repeated in two orthogonal phase-encoding directions to exclude artifact. After a minimum of 30 minutes, rest perfusion imaging was carried out at the same slice positions.

### Image analysis

Ventricular volumes, function, mass, and ejection fraction were measured using a semi-automated threshold-based technique (CMRtools, Cardiovascular Imaging Solutions, London). All volume and mass measurements were indexed to body surface area [32]. End-diastolic LV wall thickness was determined for each of the 17 American Heart Association (AHA) segments excluding the apex [33]. Late enhancement was dichotomously assessed for each segment by an expert reader blinded to the perfusion data and considered to be present if there was an area of high signal intensity on a background of adequately nulled myocardium present in two orthogonal phase-encoding directions [12].

### Perfusion analysis

**Figure 1:**
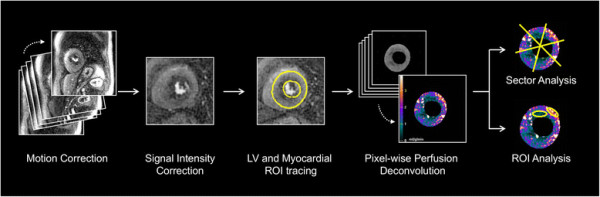
Summary of the steps involved in pixel-wise perfusion quantification and analysis.

Absolute MBF was quantified pixel-wise at rest and at peak stress as outlined in Figure 1 and as previously described [23]. In brief, endocardial and epicardial borders of the LV myocardium were manually traced using Argus CMR software (Syngo, Siemens Healthcare, Erlangen, Germany) to define myocardial regions-of-interest (ROI). Custom image processing software developed in the Interactive Data Language (Exelis Visual Information Solutions, Boulder, Colorado, USA) was used to correct surface coil-intensity bias and motion artifacts for each image series to ensure frame-to-frame correspondence of pixels. MBF was then quantified pixel-wise using model-constrained deconvolution as previously validated [23].

To avoid potential underestimation of the severity of perfusion defects in a sector-wise analysis, ROI analysis was performed using the MBF pixel maps to compare hypoperfused areas and remote hyperemic areas at stress to corresponding areas at rest. Hypoperfused areas were defined as areas which visually appeared to have the worst perfusion on stress perfusion pixel maps. Based on the minimum myocardial perfusion reserve index (MPRI = stress MBF/rest MBF) as measured from these ROI, the cohort was divided into two groups for further comparison: severe microvascular dysfunction (defined as minimum MPRI < 1.0) and non-severe groups (minimum MPRI ≥ 1.0).

To assess the relationship between perfusion, wall thickness, and the presence of late enhancement, the myocardium was also divided into 16 segments according to the 17-segment AHA model, omitting the apex. Segments were further divided into endocardial and epicardial layers to assess for transmural perfusion gradients [33]. Perfusion in areas of LGE was also compared with that in remote areas free of enhancement.

### Statistical analysis

Continuous variables are expressed as mean ± standard deviation (SD) for normally distributed variables and as medians with interquartile ranges for non-parametric data. The Kolmogorov-Smirnov test was used together with histograms to assess the normality of continuous data. Differences between parametric continuous variables were assessed using Student’s t-test, and for non-parametric data, the Mann–Whitney U-test. Categorical data are presented as frequencies and percentages. Differences between categorical variables were assessed using the χ^2^ and Fisher’s exact tests as appropriate.

To take into account correlation of repeated measurements and clustering of data from ROI and sectors within slices and patients, the perfusion data was analysed using a multilevel linear mixed effects model with patients treated as a random intercept. The relationship between the presence of LGE and perfusion was assessed using binary logistic regression together with a mixed effects multilevel generalised linear model. Two-tailed values of P < 0.05 were considered significant. Statistical analysis was performed using Stata SE Version 12 (StataCorp, College Station, Texas, USA).

## Results

### Study population

The final study cohort was comprised of 35 patients (Table 1). One patient was excluded due to an inadequate vasodilator response to adenosine, which on further questioning was due to caffeine ingestion, leaving a final cohort of 35 patients. In response to adenosine, resting heart rate rose from a mean of 69.7 ± 10.3 to 92.6 ± 13.6 beats per minute with a small fall in mean arterial pressure from 94.8 ± 11.7 to 90.3 ± 11.0 mmHg. Six out of 560 segments were excluded from analysis due to imaging artifact, encroachment of the left ventricular outflow tract on the basal slice, or problems with surface coil intensity normalisation. Overall, 99 segments had LGE.

**Table 1:** Baseline clinical and demographic characteristics of the study cohort stratified according to the presence/absence of severe microvascular dysfunction

	**Non-severe**	**Severe**	**All patients**	
**Characteristic – n (%)**	**24 (68.6)**	**11 (31.4)**	**35**	**P value**
Median age – years (IQR)	58.9 (52.3, 67.5)	52.0 (42.5, 63.6)	57.1 (48.5, 66.6)	0.256
Male sex – n (%)	18 (75.0)	7 (63.6)	25 (71.4)	0.490
Apical phenotype – n (%)	4 (16.7)	2 (18.2)	6 (17.1)	0.912
Risk factors for SCD				
Sustained VT/VF – n (%)	0 (0)	1 (9.1)	1 (2.9)	0.134
Family history of SCD – n (%)	2 (8.3)	1 (9.1)	3 (8.6)	0.941
Wall thickness ≥30 mm – n (%)	1 (4.2)	1 (9.1)	2 (5.7)	0.560
Resting LVOT obstruction ≥30 mmHg – n (%)	1 (4.2)	3 (27.3)	4 (11.4)	0.046
Non-sustained VT – n (%)	3 (12.5)	0 (0)	3 (8.6)	0.220
Unexplained syncope – n (%)	3 (12.5)	1 (9.1)	4 (11.4)	0.769
Number of risk factors for SCD – n (%)				
0	14 (58.3)	5 (45.5)	19 (54.3)	0.296
1	10 (41.7)	5 (45.5)	15 (42.9)	
2+	0 (0.0)	1 (9.1)	1 (2.9)	
NYHA functional class – n (%)				
I	16 (66.7)	8 (72.7)	24 (68.6)	0.720
II	8 (33.3)	3 (27.3)	11 (31.4)	
III	0 (0)	0 (0)	0 (0)	
IV	0 (0)	0 (0)	0 (0)	
Medications at baseline – n (%)				
β-blocker	14 (58.3)	6 (54.6)	20 (57.1)	0.833
Ca^2+^-channel blocker	0 (0)	1 (9.1)	1 (2.9)	0.134

QR = Interquartile Range; SCD = Sudden Cardiac Death; VT = Ventricular Tachycardia; VF = Ventricular Fibrillation; LVOT = Left Ventricular Outflow Tract; NYHA = New York Heart Association.

**Figure 2:**
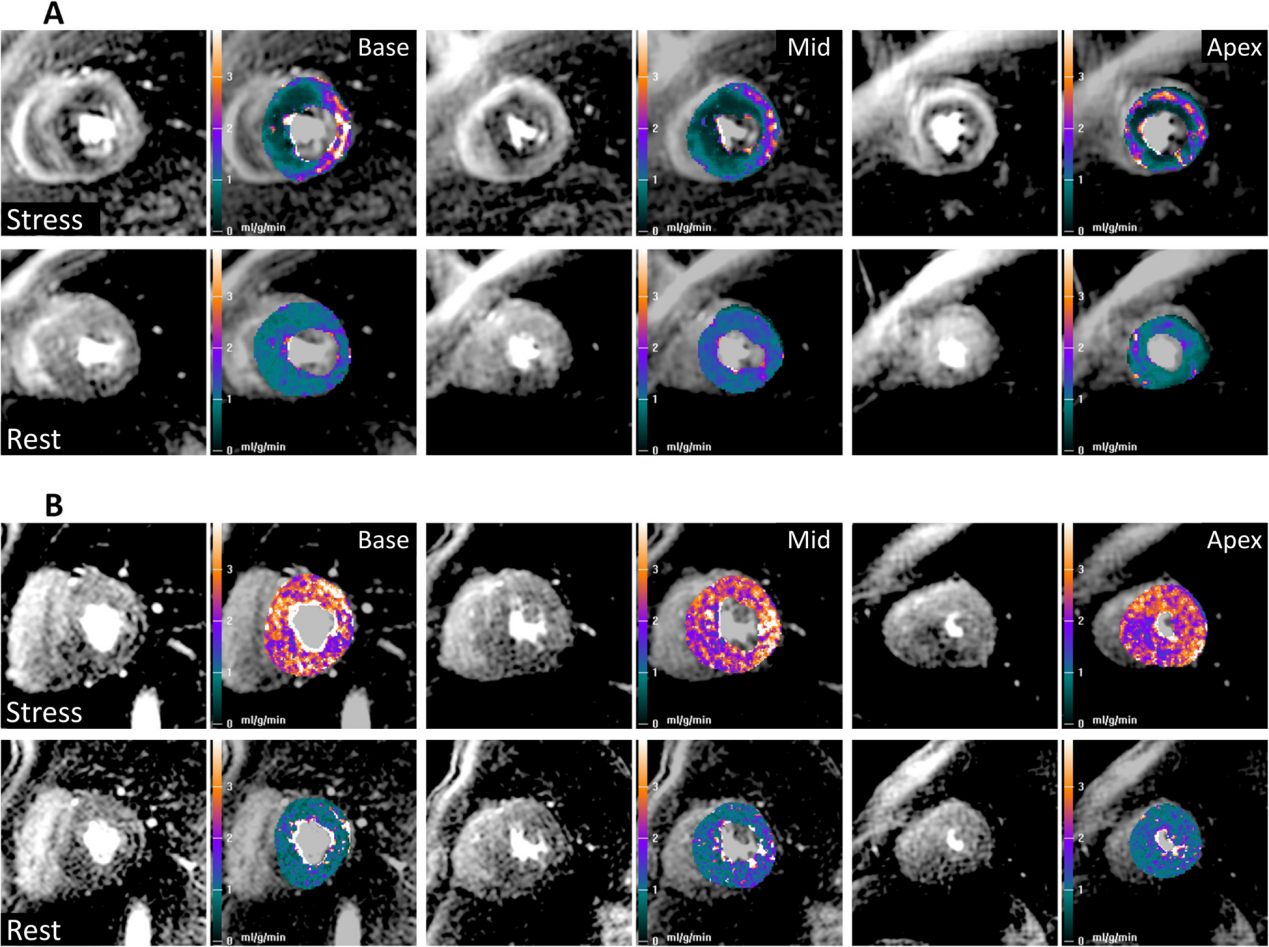
**Examples of results of pixel-wise quantitative first-pass cardiovascular magnetic resonance perfusion imaging (ml/g/min) for (A) severe microvascular dysfunction and (B) non-severe patients.** Stress images are shown on the top row and rest images on the bottom row for identical basal, mid-ventricular and apical slices together with their corresponding pixel maps.

Examples of perfusion pixel maps for the severe microvascular dysfunction and the non-severe patients are presented in Figure 2. Based on ROI analysis of the most significant perfusion defects seen in each patient, 11 (31.4%) patients showed evidence of severe microvascular dysfunction. There were no significant differences between the severe and non-severe groups with respect to baseline clinical and demographic features, although there was a trend towards a higher incidence of left ventricular outflow tract obstruction amongst those with severe microvascular dysfunction (Table 1). However, with respect to the CMR findings, the maximum end-diastolic wall thickness was significantly higher in the severe microvascular dysfunction patients versus the non-severe patients (Table 2).

**Table 2:** Baseline cardiovascular magnetic resonance findings for the study cohort stratified according to the presence/absence of severe microvascular dysfunction

	**Non-severe**	**Severe**	**All patients**	
**CMR parameters – n (%)**	**24 (68.6)**	**11 (31.4)**	**35**	**P value**
Maximum end-diastolic wall thickness – mm	18.9 ± 4.5	22.2 ± 4.0	19.9 ± 4.6	0.048
LV-EDV index – ml/m^2^	68.5 ± 15.1	64.3 ± 10.5	67.2 ± 13.8	0.419
LV-ESV index – ml/m^2^	19.5 ± 7.9	17.2 ± 6.1	18.8 ± 7.4	0.393
LV ejection fraction – %	72.0 ± 8.3	73.8 ± 6.5	72.5 ± 7.7	0.515
LV mass index – g/m^2^	93.6 ± 24.9	103.4 ± 30.0	96.7 ± 26.6	0.318

All values mean ± SD. CMR = Cardiovascular Magnetic Resonance; LV = Left Ventricular; EDV = End-diastolic volume; ESV = End-systolic volume.

### Severity of perfusion abnormalities

**Figure 3:**
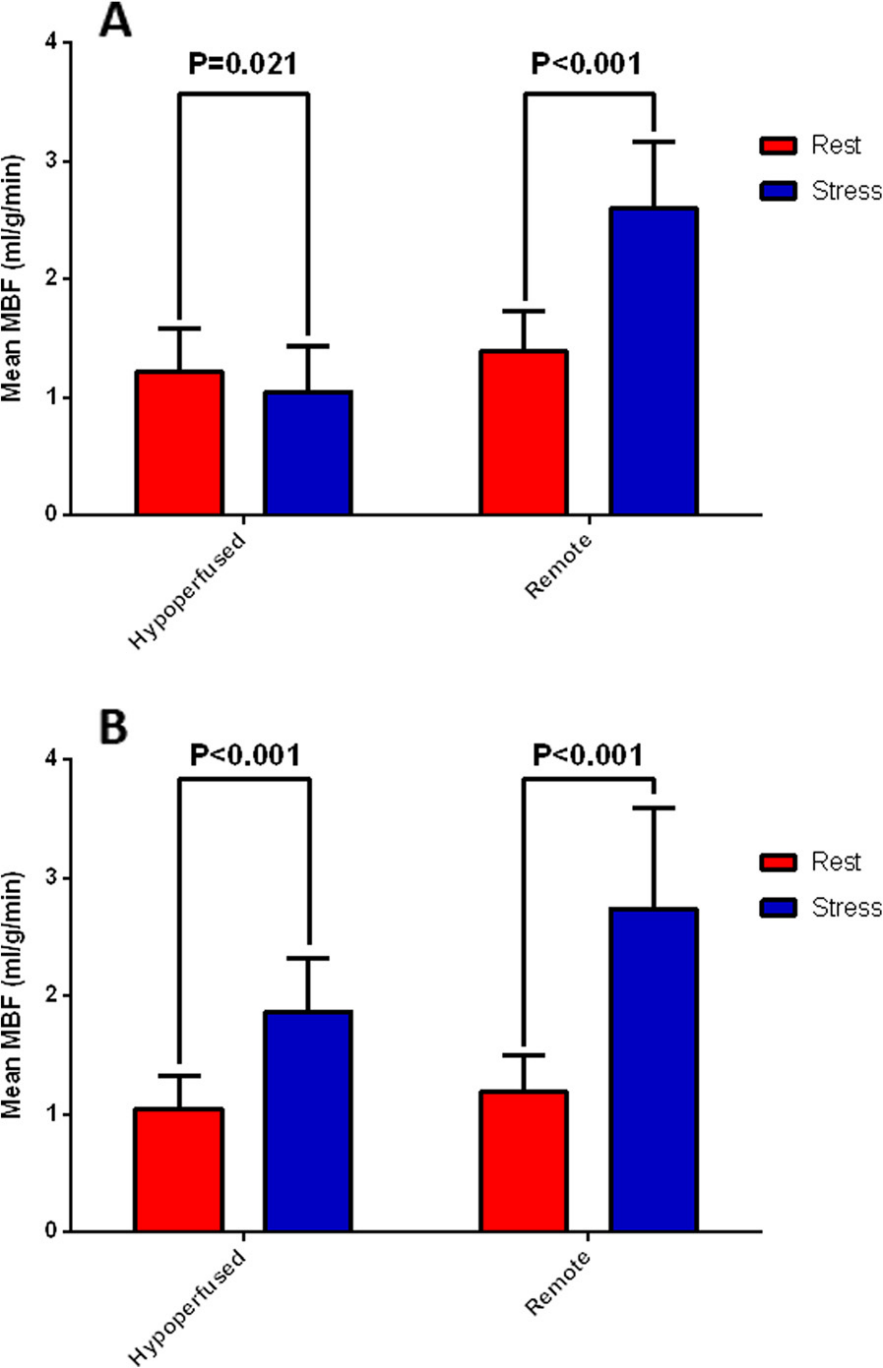
Mean myocardial blood flow (MBF) at rest and stress for hypoperfused and remote regions of myocardium for the (A) severe microvascular dysfunction and (B) non-severe patients.

ROI analysis of hypoperfused areas in the severe microvascular dysfunction patients (Figure 3A) revealed that mean resting MBF was significantly lower in hypoperfused areas relative to remote areas from the same slices (1.22 ± 0.36 ml/g/min versus 1.39 ± 0.34 ml/g/min, P < 0.001). After stress, whereas MBF rose significantly in the remote areas (1.39 ± 0.34 ml/g/min rising to 2.60 ± 0.57 ml/g/min, P < 0.001), stress MBF in hypoperfused ROI in patients with severe microvascular dysfunction not only failed to rise, but fell significantly from baseline values (1.22 ± 0.36 ml/g/min falling to 1.05 ± 0.39 ml/g/min, P = 0.021). In contrast, for the non-severe patients (Figure 3B), there was a significant rise in mean MBF with stress, even in the hypoperfused areas (1.05 ± 0.28 ml/g/min rising to 1.87 ± 0.45 ml/g/min, P < 0.001). ROI analysis in both groups showed significant hyperemic responses in regions remote from perfusion defects (1.39 ± 0.34 ml/g/min rising to 2.60 ± 0.57 ml/g/min, P < 0.001; and 1.20 ± 0.31 ml/g/min rising to 2.74 ± 0.85 ml/g/min, P < 0.001, respectively). The ratio of the minimum MPRI for hypoperfused areas to the maximum MPRI in remote regions was significantly lower in the severe microvascular dysfunction patients at 0.31 ± 0.10 versus 0.58 ± 0.16 in the non-severe group (P < 0.001).

### Transmural distribution of blood flow in response to vasodilator stress

**Figure 4:**
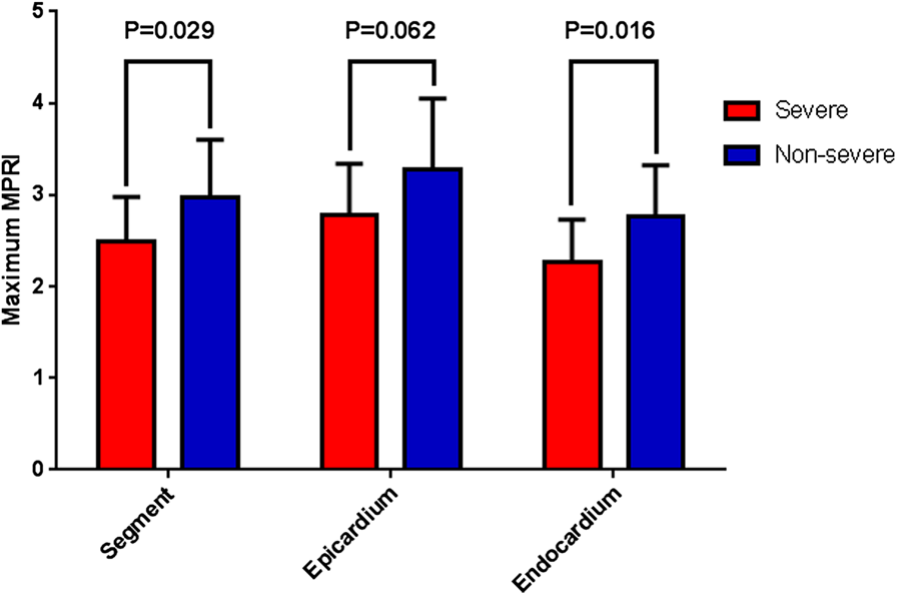
Myocardial perfusion reserve index (MPRI) in the severe and non-severe microvascular dysfunction groups for the most-hyperemic transmural segments, sub-epicardial, and sub-endocardial sub-sectors.

Adenosine achieved hyperemia, with MBF rising significantly between rest and stress. When considering the most normal sectors in each of the patients, the mean MPRI was significantly lower in the severe versus non-severe patients for sectors as a whole and in endocardial sub-sectors, with a trend towards significance in the epicardium (Figure 4). When comparing the 16 segments in all the patients, MBF rose significantly with stress in whole sectors (1.22 ± 0.34 ml/g/min rising to 2.22 ± 0.76 ml/g/min, P < 0.001) and in both endocardial and epicardial subsectors (1.25 ± 0.35 ml/g/min rising to 2.00 ± 0.76 ml/g/min, P < 0.001; and 1.20 ± 0.35 ml/g/min rising to 2.36 ± 0.83 ml/g/min, P < 0.001, respectively). At rest, endocardial MBF was significantly higher than epicardial MBF (1.25 ± 0.35 ml/100 g/min versus 1.20 ± 0.35 ml/g/min, P < 0.001) with an endocardial to epicardial MBF ratio of 1.05 ± 0.11. However, at stress, this pattern was reversed with endocardial MBF increasing significantly less than epicardial MBF (2.00 ± 0.76 ml/g/min versus 2.36 ± 0.83 ml/g/min, P < 0.001) giving a ratio of 0.85 ± 0.18. The more blunted hyperemic response in the endocardium relative to the epicardium was also reflected by a significantly lower mean MPRI (1.68 ± 0.65 versus 2.06 ± 0.73, P < 0.001 respectively).

### Perfusion and LV wall thickness

There was no significant relationship between resting MBF and sector end-diastolic wall thickness. However, at stress, there was a negative correlation between the two (β = −0.047 ml/g/min per mm, 95% confidence interval [CI]: −0.057 to −0.038, P < 0.001) with similar falls in the endocardium and the epicardium (β = −0.048 ml/g/min per mm, 95% CI: −0.058 to −0.039, P < 0.001 and β = −0.048 ml/g/min per mm, 95% CI: −0.058 to −0.038, P < 0.001, respectively).

### Perfusion and late gadolinium enhancement

**Figure 5:**
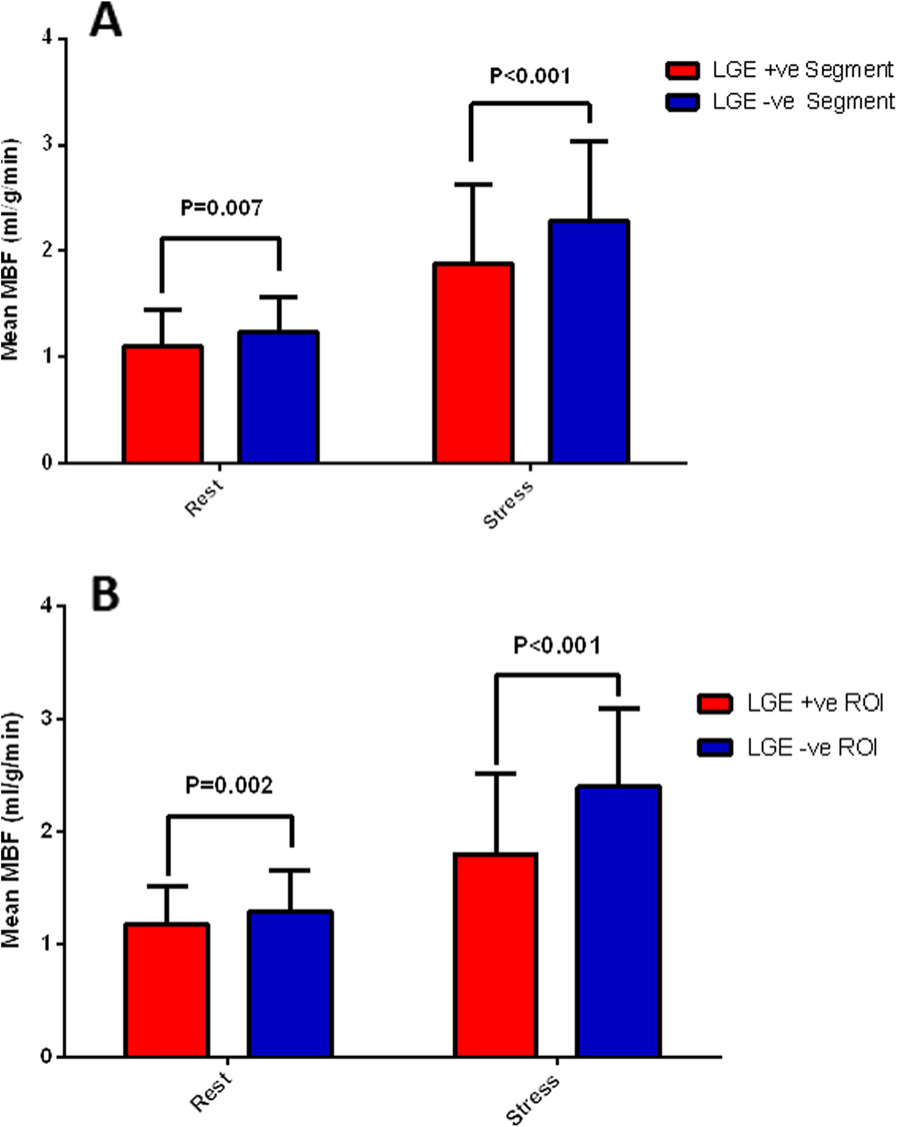
Mean myocardial blood flow (MBF) in (A) segments and (B) regions of interest (ROI) with and without late gadolinium enhancement (LGE).

Segments with LGE were significantly associated with lower perfusion at rest (odds ratio [OR] per ml/g/min increase in MBF: 0.086, 95% CI: 0.078 to 0.095, P = 0.003). This relationship remained consistent at stress (OR: 0.086, 95% CI: 0.081 to 0.092, P < 0.001) and when examined in relation to MPRI (OR: 0.053, 95% CI 0.032 to 0.089, P = 0.015). Both rest and stress MBF appeared to be significantly lower in segments with LGE relative to those without (Figure 5A), resulting in a significant difference in MPRI between segments with and without LGE (MPRI: 1.80 ± 0.74 versus 1.92 ± 0.64, P < 0.001 respectively). However, these differences did not persist after adjusting for differences in wall thickness.

ROI analysis on pixel-level perfusion maps revealed a similar, yet stronger trend of reduced MBF when comparing areas of LGE to remote areas free of LGE. This trend was observed for both rest and stress (Figure 5B). As a result, the MPRI was significantly lower in ROI with LGE than in remote areas (1.61 ± 0.65 versus 1.92 ± 0.54, P < 0.001).

## Discussion

We found evidence of widespread microvascular dysfunction in a cohort of patients with HCM being studied with multiparametric CMR. To our knowledge, this is the first study to assess stress myocardial perfusion in HCM with CMR using pixel-wise quantification techniques. This allowed us to examine perfusion abnormalities at an unprecedented level of detail. In a subset of patients, pixel-wise analysis revealed regions with not only a blunted or inadequate hyperemic response to vasodilator stress, but evidence of severe microvascular dysfunction that is likely to result in ischemia, with stress MBF levels below those of rest perfusion.

In keeping with earlier work, we found that resting endocardial MBF was significantly higher than epicardial MBF [18]. This may reflect the higher systolic wall tension and consequent higher metabolic requirements experienced by the endocardium [34],[35]. However, this transmural gradient of perfusion reversed with vasodilator stress to the detriment of the endocardium. The transmural redistribution of perfusion with stress is likely to be due to a combination of the effects of abnormal vascular resistance secondary to arteriolar medial hypertrophy and intimal hyperplasia; higher extravascular compressive forces within the endocardium; and abnormal autoregulation in response to vasodilator stress [34],[36],[37]. In support of this, as in Petersen *et al.*[18], we noted a significant relationship between wall thickness and perfusion, however, abnormalities in perfusion were also found in areas with normal wall thickness suggesting that abnormalities in vascular structure and vasomotor function may play a pre-eminent role in the genesis of microvascular ischemia in HCM [18],[38],[39]. This is in contrast to the situation with secondary LVH where extravascular factors may play a more predominant role [15],[40].

High spatial resolution non-invasive first-pass perfusion CMR techniques therefore provided valuable insights into transmural patterns of MBF, which have hitherto only been possible using microspheres, restricting such work to research in animal models [41].

Layer or sector-based analysis of MBF responses to adenosine in HCM, both in the present study and in previous work, have revealed markedly impaired vasodilator reserve. In contrast to previous work [18], pixel-wise analysis revealed evidence of severe microvascular dysfunction in hypoperfused areas, with MBF at stress actually falling below baseline resting values. This implies that there are regions in which structural abnormalities in the microvasculature and abnormal vasomotion engender vulnerability or lead to frank ischemia.

Severe microvascular dysfunction was identified only in 31% of patients, potentially identifying a higher risk sub-group of patients at risk of future adverse cardiac events. In a similar cohort of low risk and minimally symptomatic HCM patients, using PET perfusion imaging, Cecchi *et al.* identified a stress MBF of 1.10 ml/g/min as the threshold best predictive of future risk [19]. Intriguingly, the mean stress MBF of patients with severe microvascular dysfunction in the present study was 1.05 ml/g/min and the prevalence of severe microvascular dysfunction mirrored the rate of adverse cardiac events observed over long-term clinical follow-up by Cecchi *et al.*[19].

Myocardial ischemia has been proposed as a progenitor of replacement fibrosis which can be detected by the LGE technique and on histology [4],[6]. In keeping with the findings of Petersen *et al.*[18], and work by Sogtia *et al.* using PET-perfusion imaging in concert with LGE-CMR [22], we found a strong inverse association between the presence of LGE and hyperemic MBF both with ROI and sector-based analyses. In contrast to Petersen *et al.* but in agreement with Knaapen *et al.*[21], we also found reductions in resting MBF in sectors with LGE. However, neither stress nor rest differences persisted after adjusting for differences in wall thickness. Thus, while there is a strong association between impaired hyperemic MBF and the presence of LGE, this may be confounded by local disease severity as indirectly reflected by local wall thickness. On histology, Varnava *et al.* found a poor interrelationship between myocardial fibrosis, small vessel disease and disarray [16]. These findings are also in accord with those of Tyan *et al.* who semi-quantitatively assessed the distribution of perfusion abnormalities and tissue injury using CMR [42]. The absence of a dose–response relationship between reduced hyperemic MBF and the observed spatial distribution of LGE suggests that factors other than or beyond ischemia must be implicated in the pathogenesis of replacement fibrosis in HCM. Although the sub-endocardium is most severely affected by microvascular ischemia, paradoxically, the overwhelming preponderance of LGE is seen in a mid-wall distribution, typically sparing the sub-endocardium [14],[43]. This implies that factors beyond microvascular ischemia, possibly under genetic or epigenetic control significantly modulate the development of replacement fibrosis in HCM. Further work is required to delineate the interrelationships of fibrosis and microvascular dysfunction in-vivo, and in particular, their temporal relationship.

### Limitations

The population studied was drawn from referrals to our clinical service which is a tertiary center, leading to potential selection bias towards higher risk cases. However, patients with implantable cardioverter defibrillators (ICDs) who have been deemed high risk will have been excluded due to the contraindication of CMR in this group potentially counterbalancing this. In addition, 97% of patients had 0 or only 1 risk factor for SCD.

Myocardial fibrosis was assessed using the LGE technique. Whilst this detects replacement fibrosis, it does not allow the quantification of interstitial fibrosis [44]. The association between fibrosis and perfusion abnormalities may therefore have been underappreciated. Nevertheless, replacement fibrosis is thought to be driven by ischemic necrosis and is the distinct type of fibrosis that has been most clearly associated with myocardial ischemia in HCM [4],[5],[24]. Future developments in interstitial imaging using T1-mapping techniques may allow the relationship between interstitial fibrosis, total fibrotic burden and perfusion to be addressed [44].

Finally, we were unable to determine the prognostic significance of our findings given our limited sample size and the relatively low event rate seen in HCM [45]. Nevertheless, our finding of severe microvascular dysfunction in a subgroup of patients with HCM warrants further investigation to determine the potential utility of this phenomenon for risk stratification.

## Conclusions

In summary, coronary microvascular dysfunction is a common finding in HCM and is associated with increasing wall thickness and with the presence of LGE. Fully quantitative pixel-wise first-pass CMR perfusion imaging identifies a significant number of patients with localised severe microvascular dysfunction that is likely to result in ischemia. Further work is required to determine if this phenomenon heralds an increased risk of future adverse cardiovascular events.

## Competing interests

Professor Dudley J Pennell is a consultant to Siemens and a director of Cardiovascular Imaging Solutions. The Royal Brompton Hospital has research collaboration agreements with Siemens.

## Authors’ contributions

TFI, LYH, DF, DJP, SKP, and AEA were involved in the conception and design of the study as well as data collection, analysis, interpretation, and drafting of the manuscript. TFI, LYH, AMG, CG, BH, NM, RW, PF, and PG were involved in data gathering, analysis, interpretation, protocol development, drafting and revision of the manuscript. AJ, AG and ROH were involved in data analysis, drafting of the manuscript and in its revision for important content. MR performed all statistical analysis. All authors critically revised the manuscript for important intellectual content, read and approved the final manuscript.
